# Interferon Epsilon-Mediated Antiviral Activity Against Human Metapneumovirus and Respiratory Syncytial Virus

**DOI:** 10.3390/vaccines12101198

**Published:** 2024-10-21

**Authors:** Iván Martínez-Espinoza, Pius I. Babawale, Hannah Miletello, Nagarjuna R. Cheemarla, Antonieta Guerrero-Plata

**Affiliations:** Department of Pathobiological Sciences, School of Veterinary Medicine, Louisiana State University, Baton Rouge, LA 70803, USA

**Keywords:** interferon epsilon, IFN-ε, respiratory, lung, HMPV, RSV, epithelial cells, NHBE cells, ALI culture

## Abstract

Background: Interferon epsilon (IFN-ε) is a type I IFN that plays a critical role in the host immune response against pathogens. Despite having demonstrated antiviral activity in macrophages and mucosal tissues such as the female reproductive tract and the constitutive expression in mucosal tissues such as the lung, the relevance of IFN-ε against respiratory viral infections remains elusive. Results: We present, for the first time, the expression of IFN-ε in alveolar epithelial cells and primary human bronchial epithelial cells grown in an air–liquid interface (ALI) in response to human metapneumovirus (HMPV) and respiratory syncytial virus (RSV) infection. The molecular characterization of the IFN-ε induction by the viruses indicates that the expression of RIG-I is necessary for an optimal IFN-ε expression. Furthermore, treatment of the airway epithelial cells with rhIFN-ε induced the expression of IFN-stimulated genes (ISGs) and significantly restricted the viral replication of HMPV and RSV. Conclusions: These findings underscore the relevance of IFN-ε against viral infections in the respiratory tract.

## 1. Introduction

The interferon (IFN) family is divided into three main subfamilies. The type I IFN subfamily includes IFN-α (with 13 subtypes), IFN-β, and other less known members such as IFN-ε, IFN-κ, and IFN-ω. The type II IFN subfamily is represented only by IFN-γ, and the type III IFN subfamily comprises IFN-λ1 (IL-29), IFN-λ2 (IL28A), IFN-λ3 (IL-28B), and the lesser known IFN-λ4 [1]. Interferon epsilon (IFN-ε) was initially characterized in mice and humans in 2004 [2]. The constitutive expression of IFN-ε is primarily detected in the uterus, cervix, vagina, and ovaries [3,4,5,6]. However, mouse studies reveal that IFN-ε is also detectable in several other tissues, including the lungs [2]. Recent work has shown a constitutive expression of IFN-ε in mouse bronchial epithelial cells [7]. In *Rhesus macaques*, IFN-ε has also been found to be constitutively expressed in the epithelial cells lining the bronchioles [8]. Nevertheless, the antiviral role of IFN-ε in the respiratory epithelium remains elusive.

In addition to its distinct characteristics, IFN-ε has demonstrated antiviral activity both in vitro and in vivo against several viruses, particularly in the female genitourinary tract. It has been observed that IFN-ε can control herpes simplex virus 2 (HSV-2) [6] and impair Zika virus (ZIKV) replication in human vaginal epithelial cells via the induction of IFN-stimulated genes (ISGs) [9]. IFN-ε has also been reported to suppress human immunodeficiency virus (HIV) replication at multiple stages of infection in peripheral blood lymphocytes [10] and human macrophages [11]. Despite the importance of IFN-ε in controlling these viral infections and its presence in the airway epithelium [7,8], there are no reports addressing its role in respiratory viruses. Consequently, the role of IFN-ε in host defense against respiratory viruses remains largely unknown.

Human respiratory syncytial virus (RSV) and Human Metapneumovirus (HMPV) are enveloped, negative single-stranded RNA pneumoviruses that cause upper and lower respiratory infections [12]. They are the causative agents of mild diseases such as common cold-like symptoms, as well as more severe clinical presentations such as bronchiolitis and pneumonia, with high chances of hospitalization and, in severe cases, mortality [13,14]. Both viruses share multiple characteristics, including epidemiological aspects, with RSV and HMPV being the etiological agents responsible for respiratory tract infections in infants [15,16,17], young children [18,19,20,21,22], the elderly [23,24], and immunocompromised individuals [25,26]. The seasonal distribution appears to be similar between the two viruses, having RSV season from late fall (Oct–Nov) to early spring (Mar–Apr) and overlapping with HMPV season occurring from late winter (Jan–Feb) to May [19,27,28,29]. Globally, it is estimated that, annually, 14.2 million acute lower respiratory tract infection (LRTI) cases are attributed to HMPV, while 33 million are attributed to RSV in children under 5 years [30,31]. For both viruses, the incidence in children ranges between 5% and 15%, though some researchers have reported higher rates [15,16,19,20,32,33,34,35,36]. Despite the risk that both viruses represent, effective vaccines for children are not available to prevent HMPV or RSV infection. Therefore, there is a need to explore potential agents that can contribute to the control of these viral infections.

In the present study, we analyzed the induction of IFN-ε expression by HMPV and RSV infection in human respiratory epithelial cells and identified the contribution of RIG-I as the main cytosolic receptor responsible for its expression. Furthermore, using a cell line and an organotypic cell culture, we demonstrated the antiviral effect of IFN-ε against HMPV and RSV infections. These findings highlight the importance of IFN-ε in respiratory viral infections.

## 2. Materials and Methods

### 2.1. Cell Culture

A549 cells (ATCC CCL-185C, Manassas, VA, USA), a human cell line with characteristics of type II alveolar epithelial cells [37] derived from lung adenocarcinoma, were grown in F12K medium (Corning, Glendale, AZ, USA) supplemented with 10% FBS (Gibco, Gaithersburg, MD, USA) and 1% penicillin/streptomycin (Gibco, Gaithersburg, MD, USA), which will be referred to as the complemented medium. Cells were maintained in a 5% CO_2_ incubator at 37 °C.

LLC-MK2 cells (ATCC, CCL-7, Manassas, VA, USA) and HEp-2 cells (ATCC CCL 23, Manassas, VA, USA) were cultured in MEM/EBSS medium (HyClone, Logan, UT, USA) supplemented with 10% FBS (Gibco, Gaithersburg, MD, USA) and 1% penicillin-streptomycin (Gibco, Gaithersburg, MD, USA), herein referred to as the complemented medium.

Normal human bronchial epithelial (NHBE) cells were purchased from Lonza (Lonza Bioscience, Walkersville, MD, USA). The cells were seeded on 0.4 μm transwell cell culture inserts in Pneumacult Ex Plus Basal medium (STEMCELL Technologies, Cambridge, MA, USA) at 37 °C under a 5% CO_2_ atmosphere. After the cells reached 100% confluency, they were airlifted in an air–liquid interface (ALI) culture system. The basal medium was substituted with Pneumacult ALI complete medium (STEMCELL Technologies, Cambridge, MA, USA). A fully differentiated pseudostratified ALI culture tissue was established at 4 weeks post-airlift.

### 2.2. Virus Stocks

The HMPV strain CAN97-83 was obtained from the Centers for Disease Control, USA. HMPV CAN97-83 expressing the green fluorescent protein (HMPV-GFP) was purchased from Vira Tree (Vira Tree LLC, Research Triangle Park, NC, USA). Both viruses were propagated in LLC-MK2 cells in MEM containing 1 μg trypsin/mL (Worthington Biochemicals, Lakewood, NJ, USA) and purified by polyethylene glycol precipitation, followed by centrifugation on a 60% sucrose cushion [38,39]. RSV strain A2 was purchased (ATCC, Manassas, VA, USA), and the recombinant RSV-A2-expressing the red fluorescent protein gene (rrRSV) was generated as we previously described [40]. Both viruses were propagated in HEp-2 cells (ATCC CCL 23, Manassas, VA, USA) and purified by polyethylene glycol precipitation, followed by centrifugation on 35% to 65% discontinuous sucrose gradients [41]. The viral passage for all viruses used in the experiments was no higher than passage 7. The viral titer (PFU/mL) for HMPV was determined by a combined method of a methylcellulose plaque assay and a cell-based immunoassay in LLC-MK2 cells, as we previously reported [40]. The viral titer (PFU/mL) for RSV A2 was determined by a methylcellulose plaque assay on HEp-2 cells [42,43]. For HMPV-GFP and rrRSV, the viral titer was expressed in fluorescent forming units per milliliter (FFU/mL) and identified by the expression of GFP or RFP in the infected cell monolayers after serial dilutions and methylcellulose overlay.

### 2.3. IFN-ε Treatment and Viral Infection

A549 cells were pretreated with IFN-ε (R&D Systems, Minneapolis, MN, USA) 24 h before infection. After that time, the cells were washed twice with PBS and infected with HMPV in the presence of 1 μg/mL of trypsin or infected with RSV in MEM medium without serum at an MOI of 1.0. After 2 h of viral adsorption, the viral inoculum was removed, and 1 mL of complemented medium was added. At 24 h post-infection, cells or cell lysates were collected for further analysis.

Fully differentiated NHBE cells in ALI cultures were pretreated with IFN-ε (R&D Systems, Minneapolis, MN, USA) 24 h before infection. The apical surface of the cultures was washed twice with PBS before infection. The cells were infected with the viruses at an MOI of 0.02 and incubated at 37 °C. The viral inoculum was removed after 2 h of adsorption, and the cells were cultured for 3 days. The infected tissues were continuously exposed to IFN-ε by the daily replenishment of the cytokine.

### 2.4. Generation of CRISPR/Cas9 Gene Knockout A549 Cells

CRISPR knockout (KO) A549 cell lines were generated using the LentiCRISPR v2 (Addgene, Watertown, MA, USA) single vector system. Sequences of single guide RNA (sgRNA) targeting MyD88, RIG-I, and MDA-5 were cloned into LentiCRISPR v2 (Addgene) via BsmBI sites. The Cloned Lentivirus plasmid was transfected together with psPAX (Addgene) and pVSV-G (Addgene) into 293FT cells using Lipofectamine™ 3000 (Invitrogen, Waltham, MA, USA). For the generation of clonal KO A549 cells, lentivirus was collected and used to transduce A549 cells with 10 µg/mL of Polybrene (Millipore Sigma, Hayward, CA, USA). Cells were selected with 10 µg/mL Puromycin (Gibco, Gaithersburg, MD, USA) for 15 days. Transduced A549 cells were seeded in limiting dilutions and individual clones were isolated, expanded, and validated by Western blot.

### 2.5. Western Blot

The validation of KO cell lines was carried out by Western blot assays. In total, 2.5 µg of the total protein from each cell lysate was used. The samples were mixed with 4X Laemmli buffer and 10% 2-mercaptoethanol (Bio-Rad Laboratories Inc., Hercules, CA, USA) and heated for 5 min. The samples were then subjected to electrophoresis in 10% acrylamide gels. Protein bands were electroblotted onto PVDF membranes (Bio-Rad Laboratories Inc, Hercules, CA, USA), followed by the treatment of the membranes with 0.5% BSA, 0.1% Triton in PBS for 1 h. Anti-RIG-I (Santa Cruz biotech, Santa Cruz, CA, USA), anti-MDA-5 (Abcam, Cambridge, MA, USA), and anti-MyD88 (Cell Signaling Technology, Danvers, MA, USA) were used as primary antibodies. Anti-rabbit IgG-HRP-linked antibody (Cell Signaling Technology, Danvers, MA, USA) was used as a secondary antibody. Membranes were revealed using the Pierce^TM^ ECL western blotting substrate (ThermoFisher Scientific, Waltham, MA, USA). The same membranes were processed using stripping buffer (ThermoFisher Scientific, Waltham, MA, USA) to analyze the housekeeping gene control GAPDH. Anti-GAPDH Rabbit mAb HRP-conjugated (ABclonal, Woburn, MA, USA) or a combination of anti-GAPDH Rabbit mAb (Sino Biological USA, Inc., Houston, TX, USA) with anti-rabbit IgG-HRP-linked antibody (Cell Signaling Technology, Danvers, MA, USA) were used. Image visualization and acquisition were carried out using X-ray film for chemiluminescence.

### 2.6. RNA Extraction and Quantitative Real-Time Reverse Transcription-PCR (RT-qPCR)

RNA was obtained with an RNeasy-plus kit (Qiagen, Germantown, MD, USA). Gene expression was quantified as follows: the first-strand cDNA was synthesized from total RNA using the LunaScript RT SuperMix Kit (New England Biolabs, Ipswich, MA, USA), according to the manufacturer’s instructions. cDNA fragments of interest were amplified using the PowerTrack SYBR Green Master Mix (ThermoFisher Scientific, Waltham, MA, USA). Primers for IFN-α, IFN-β, IFN-ε, ISG56/IFIT1, ISG54/IFIT2, ISG60/IFIT3, ISG15, MX1, IRF7, HMPV, RSV, and GAPDH were run on the QuantStudio™ 12k PCR system (Applied Biosystems, Foster City, CA, USA). All primers were purchased from Integrated DNA Technologies. The quantification of target gene expression was carried out using the CT method (ΔΔCT), normalized relative to the endogenous reference (GAPDH) expression. Absolute viral quantification (viral copies/ng) was assessed by generating a standard curve prepared with a plasmid containing the HMPV N protein gene or the RSV N protein gene. Data were analyzed using QuantStudio™ 12k Flex Software Version 1.3.

### 2.7. Flow Cytometry

The cells were detached and washed with PBS containing 0.5% BSA (PBS/BSA). Cell viability was determined by staining the cells with the live/dead dye 7-AAD, according to the manufacturer’s instructions (eBioscience, San Diego, CA, USA). Processed samples were immediately analyzed after staining. The samples were acquired on a FACScan flow cytometer (BD Biosciences, Franklin Lakes, NJ, USA). Data analysis was performed with FlowJo v10.10.0 software (BD Biosciences, Franklin Lakes, NJ, USA).

### 2.8. Live-Cell Imaging and Analysis

Infected cells with HMPV-GFP or rrRSV, in the presence or absence of rhIFN-ε, were analyzed using the Incucyte^®^ Zoom HD/2CLR time-lapse microscopy system (Sartorius, Bohemia, NY, USA). Fluorescence was acquired using acquisition times of 400 ms for the green channel (HMPV) and 800 ms for the red channel (RSV). The instrument determined the fluorescence intensity as Green Calibrated Units (GCU) × μm^2^/image or Red Calibrated Units [44] × μm^2^/image. Fluorescence intensity was further calculated as a percentage from the infected untreated cells.

### 2.9. Statistical Analysis

Statistical analyses were calculated by an unpaired student *t*-test and one-way analysis of variance (ANOVA) to ascertain the differences between the tested conditions, followed by post-tests to correct for multiple comparisons using GraphPad Prism 10.3.1 (GraphPad Software, San Diego, CA, USA). The results are expressed as means ± standard errors of the means (SEM).

## 3. Results

### 3.1. IFN-ε Is Induced by RSV and HMPV in Alveolar Epithelial Cells

**Figure 1:**
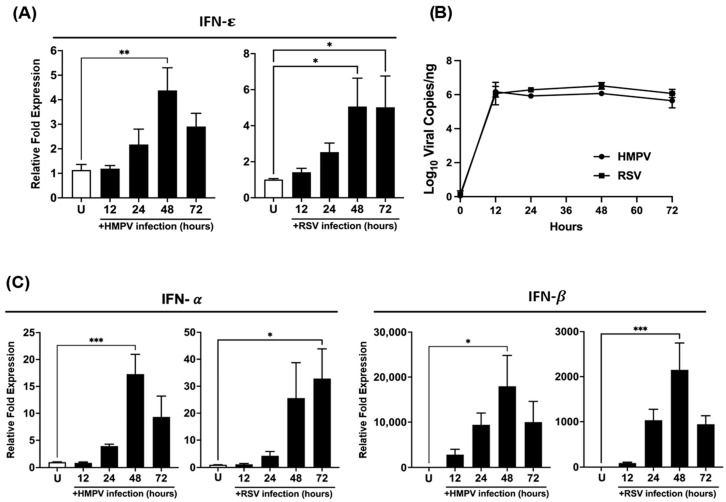
Expression of IFN-ε by HMPV in A549 cells. Cells were infected with HMPV or RSV at an MOI 1 and cultured for 72 h. Cell lysates were collected at different time points after infection and analyzed by RT-qPCR to determine (**A**) the expression of IFN-ε (n = 4); (**B**) the viral copy numbers of the N protein gene for HMPV and RSV (n = 3); and (**C**) the expression of IFN-α and IFN-β (n = 4). The bar graphs represent the mean ± standard errors of the means. Statistical differences were calculated using ANOVA followed by a Sidak’s multiple comparison test * *p* < 0.05; ** *p* < 0.01; *** *p* ≤ 0.001. Uninfected (U).

To evaluate the response of IFN-ε induced by RSV and HMPV infection, we first analyzed the expression of IFN-ε in A549 cells, a type of human alveolar basal epithelial cell line. The cells were infected with HMPV or RSV at a multiplicity of infection (MOI) of 1, and the samples were collected at 12, 24, 48, and 72 h after infection. As shown in Figure 1A, HMPV induced IFN-ε as early as 24 h, reaching a peak of induction at 48 h and a decline by 72 h. A similar induction pattern was observed when the cells were infected with RSV. However, unlike HMPV, the induction of IFN-ε by RSV was maintained at least up to 72 h, the last time point analyzed. We also observed that the levels of IFN-ε induction by both viruses were comparable, with a peak of a ~4–5-fold increase with a slightly higher expression by RSV infection. To validate the observed differences in IFN-ε induction between both viruses, we assessed a comparative analysis of the level of the viral infections by quantifying the viral copies by RT-qPCR. The data indicated that the cells were comparatively infected by each virus (Figure 1B). Furthermore, to compare the induction of IFN-ε to other type I IFNs in A549 cells, the expression of IFN-β and IFN-α was evaluated. The data shown in Figure 1C indicate that IFN-β is the highest induced by both viruses in A549 cells, where HMPV was a more potent inducer of IFN-β than RSV. We also observed the same pattern between the three IFNs regarding the HMPV infection. In the case of the RSV infection, IFN-α was still rising after 72 h, while IFN-β rapidly decreased and IFN-ε remained stable in a plateau.

### 3.2. RIG-I Contributes to the Induction of IFN-ε by RSV and HMPV

**Figure 2:**
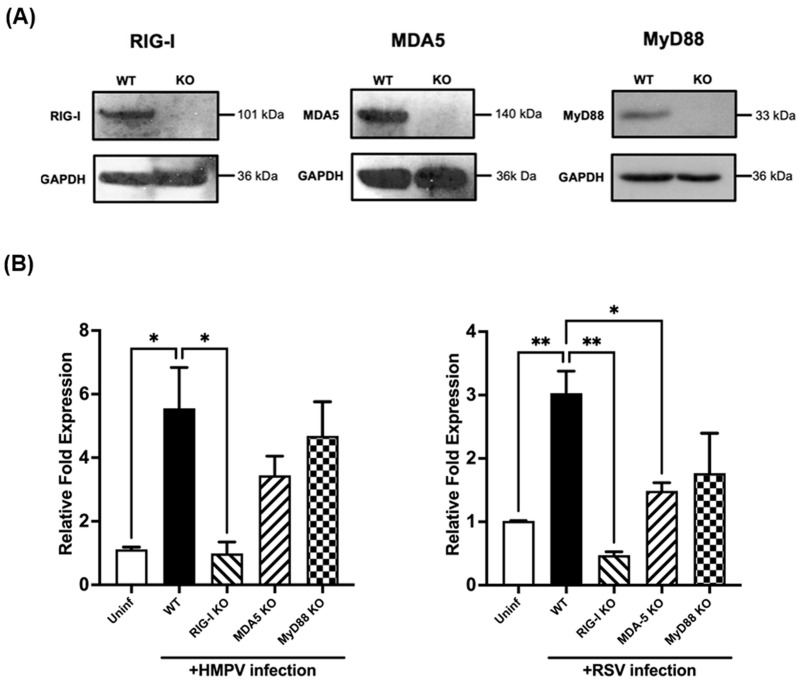
Role of PRRs in IFN-ε expression by RSV and HMPV. (**A**) Western blot of KO A549 cell lines lacking MyD88, RIG-I, or MDA-5. GAPDH was used as a housekeeping gene control. (**B**) KO A549 cells were infected with HMPV or RSV at an MOI of 1.0 for 48 h. Cell lysates were collected and analyzed for IFN-ε. Fold Increase Expression was determined by RT-qPCR. The bar graphs represent the mean ± standard errors of the means (n = 3). Statistical differences were calculated using ANOVA followed by a Tukey’s multiple comparison test. * *p* < 0.05, ** *p* < 0.01.

Next, we investigated the activation pathway involved in the induction of IFN-ε by RSV and HMPV. The contribution of pattern recognition receptors (PRRs) such as RIG-I, MDA5, and Toll-like receptors (TLRs) was analyzed. We generated KO A549 cell lines for RIG-I, MDA-5, and MyD88, as described in the methods. RIG-I and MDA5 are the main RIG-I-like receptors (RLRs) [45]. MyD88 was chosen because of its critical role as the adaptor molecule in the activation of most TLRs. KO cell lines were selected from a single-cell clone, propagated, and further validated through Western Blot assays. As shown in Figure 2A (and Supplementary Materials), the expression of RIG-I, MDA5, and MyD88 was assessed, demonstrating that the KO cell lines did not express the target protein when compared to wild-type cells [46]. GAPDH was used as a loading internal control. Once the KO cell lines were validated, KO and WT cells were infected with HMPV or RSV at an MOI of 1 for 48 h, where we previously observed the peak of IFN-ε induction (Figure 1A). The expression of IFN-ε was analyzed by RT-qPCR. Our results demonstrated that RIG-I is a key RLR contributing to the IFN-ε induction by HMPV, while no statistical changes were found in the absence of MDA5. However, for RSV, we observed a significant contribution of both RIG-I and MDA-5 in the expression of IFN-ε, with a more prominent role for RIG-I. The contribution of MyD88 in the IFN-ε expression by either virus was not statistically significant.

### 3.3. IFN-ε Induces the Expression of ISGs

**Figure 3:**
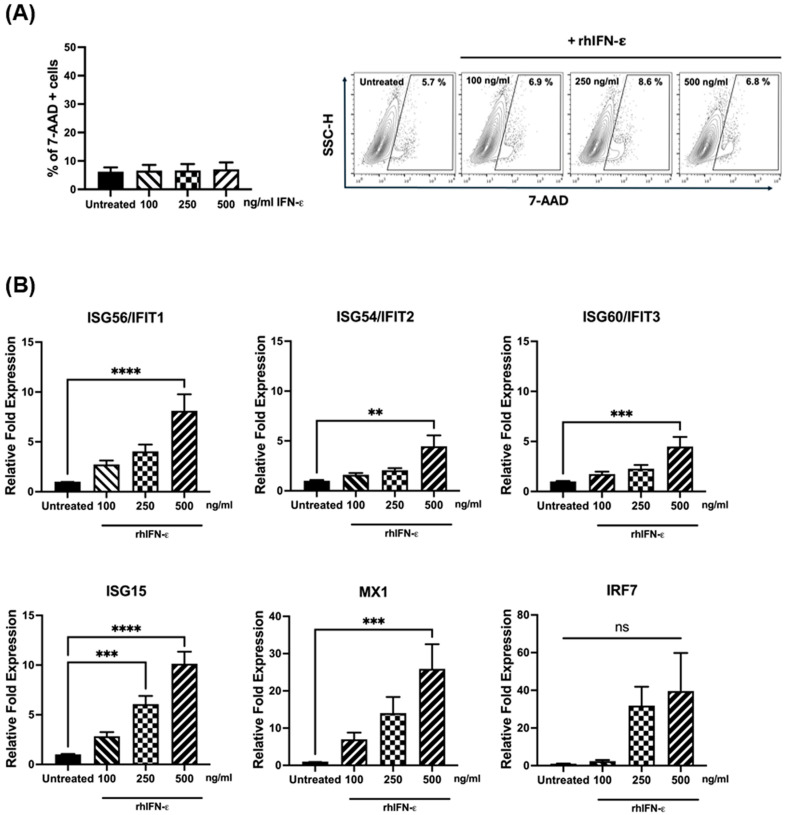
Induction of ISGs in A549 cells. Cells were treated with increasing concentrations of rhIFN-ε for 24 h. (**A**) To determine cell viability, cells were collected and stained with 7-AAD and analyzed by flow cytometry. The bar graphs show the percentage of 7-AAD-positive cells. Contour plots show representative data of 7-AAD positive cells. (**B**) Relative expression of ISGs was assessed with RT-qPCR and normalized to GAPDH. The bar graphs represent the mean ± standard errors of the means (n = 3). Statistical differences were calculated using ANOVA followed by Dunnett’s multiple comparison test. ** *p* < 0.01; *** *p* < 0.001; **** *p* ≤ 0.0001. Non-significant (ns).

The antiviral response exerted by IFNs includes the induction of the expression of IFN-stimulated genes (ISGs) [47,48], which interfere with the viral replication through multiple mechanisms [47,48]. Here, we first determined the cytotoxic effect of recombinant human IFN-ε. A549 cells were treated with IFN-ε at concentrations of 100, 250, and 500 ng/mL. After 24 h, cell damage was analyzed through flow cytometry by quantifying the overexpression of 7-AAD, a fluorescent compound that is membrane-impermeant and does not bind to viable cells. As shown in Figure 3A, we observed a percentage of 5.7 of 7AAD positive cells in the untreated cells, which did not significantly change upon treatment with any concentration of IFN-ε used. Representative dot plots illustrate the expression of 7-AAD in the untreated and treated cells. Taken together, our data indicate that the different concentrations used did not have an impact on cell viability.

To explore the antiviral effect of IFN-ε in the alveolar cell line, the expression of ISGs was assessed. A549 cells were treated with IFN-ε, as mentioned above. After 24 h, the expression of ISG56/IFIT1, ISG54/IFIT2, ISG60/IFIT3, ISG15, MX1, and IRF7 was quantified through RT-qPCR. Our data show that at a concentration of 500 ng/mL, IFN-ε significantly induced the expression of ISG56, ISG54, ISG60, ISG15, and MX1. Although induced, IRF7 did not reach statistical significance. When testing lower concentrations of IFN-ε, we observed that ISG15 was the only one found to be significantly expressed at 250 ng/mL (Figure 3B). Overall, IFN-ε was able to induce an antiviral response with a minimal cytotoxic effect on airway epithelial cells.

### 3.4. IFN-ε Prevents the Infection and Reduces the Viral Titers of HMPV and RSV in A549 Cells

**Figure 4:**
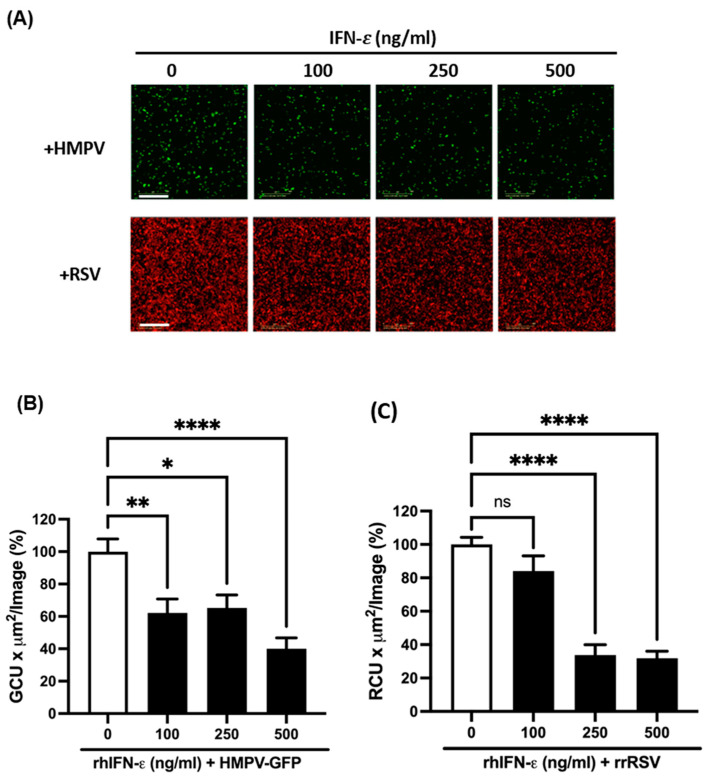
Susceptibility of RSV and HMPV infection to IFN-ε. A549 cells were treated with increasing concentrations of rhIFN-ε for 24 h followed by infection with HMPV or RSV at an MOI of 1.0 for 24 h. Susceptibility was determined by a reduction in the fluorescence signal using the incucyte^®^ system. (**A**) Representative images of fluorescence from cells infected with HMPV-GFP (upper panel) or rrRSV (lower panel). Size bar 800 μm. (**B**,**C**) Fluorescence intensity represented in a percentage from the untreated cells infected with (**B**) HMPV or (**C**) RSV. The bar graphs represent the mean ± standard errors of the means (n = 5). Statistical differences were calculated using ANOVA followed by a Dunnett’s multiple comparison test. * *p* < 0.05; ** *p* < 0.01; **** *p* ≤ 0.0001. Non-significant (ns).

To the best of our knowledge, there are no studies that have investigated the antiviral effect of IFN-ε against respiratory viruses. Therefore, we further assessed the effect of IFN-ε on its capacity to interfere with the infection of RSV and HMPV, and we infected the epithelial cells in the presence of IFN-ε. As tested above, A549 cells were pretreated with IFN-ε at concentrations of 100, 250, and 500 ng/mL for 24 h. After that time, the cells were infected with HMPV or RSV at an MOI of 1 and cultured for an additional 24 h. The presence of IFN-ε was maintained throughout the culture period. For this set of experiments, we used recombinant viruses that express GFP (HMPV-GFP) or RFP (rrRSV) in the infected cells, which facilitated the accurate evaluation of the antiviral effect of IFN-ε. The quantification of the fluorescence was assessed using the IncuCyte^®^ system and software. Figure 4 shows a reduced fluorescence signal in infected cells cultured in the presence of IFN-ε (Figure 4A). The quantification of the percentage of infected cells indicates that cells treated with IFN-ε significantly reduced HMPV infection by 37.8% ± 8.5 at a concentration of 100 ng/mL, by 34.7% ± 7.9 with 250 ng/mL, and by a maximum of 59.9% ± 8.5 when 500 ng/mL were used (Figure 4B). In the case of RSV, the decrease in the percentage of infected cells was larger than that of HMPV. We observed a reduction of 66.2% ± 6.1 and 66.8 ± 4.1 when using IFN-ε concentrations of 250 and 500 ng/mL, respectively. However, no significant effect on RSV-infected cells was observed when cells were treated with 100 ng/mL of IFN-ε (Figure 4C).

**Figure 5:**
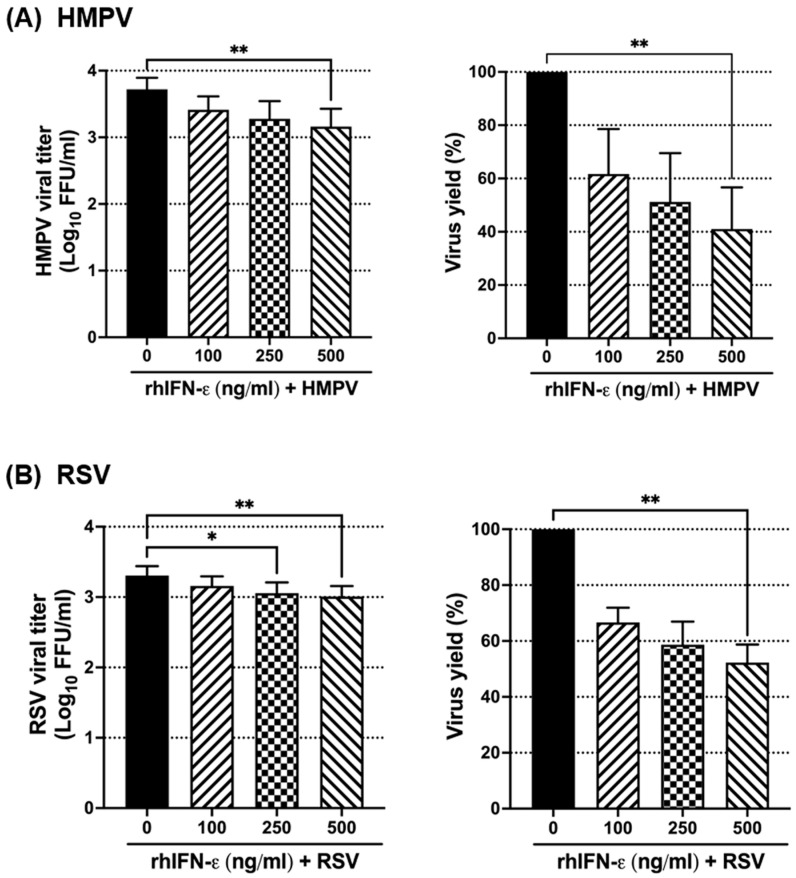
Effect of IFN-ε on RSV and HMPV infection in A549 cells. Cells were treated with increasing concentrations of rhIFN-ε for 24 h followed by infection with (**A**) HMPV or (**B**) RSV at an MOI of 1.0 for 24 h. Viral titers were quantified in cell supernatants by a plaque assay. Virus yield is expressed as a percentage relative to the untreated infected cells. The bar graphs represent the mean ± standard errors of the means (n = 4). Statistical differences were calculated using ANOVA followed by a Dunn’s multiple comparison test. * *p* < 0.05; ** *p* < 0.01.

The antiviral response of IFN-ε was additionally evaluated by assessing the release of infectious viruses from the infected cultures. A549 cells were treated with IFN-ε and infected using the same conditions as in Figure 4. Supernatants were collected after 24 h of infection, and titration of the infectious virus was evaluated by methylcellulose overlay. The results shown in Figure 5A indicate that adding IFN-ε at a concentration of 500 ng/mL significantly reduced the HMPV load by 0.55 log10 with a reduction of 60% of the viral yield. Regarding the effect of IFN-ε on RSV replication, we observed that cells treated with concentrations of 250 ng/mL and 500 ng/mL of IFN-ε significantly reduced the viral load compared to the untreated cells, resulting in a decrease of 0.25 log10 and 0.3 log10, respectively. The RSV infection was reduced between 42 and 48% at those two concentrations. No significant effect was observed at 100 ng/mL (Figure 5B). Overall, these data suggest that RSV and HMPV are differentially susceptible to the antiviral effect of IFN-ε.

### 3.5. IFN-ε Is Constitutively Expressed in Human Bronchial Epithelial Cells and Induced by HMPV and RSV Infection

**Figure 6:**
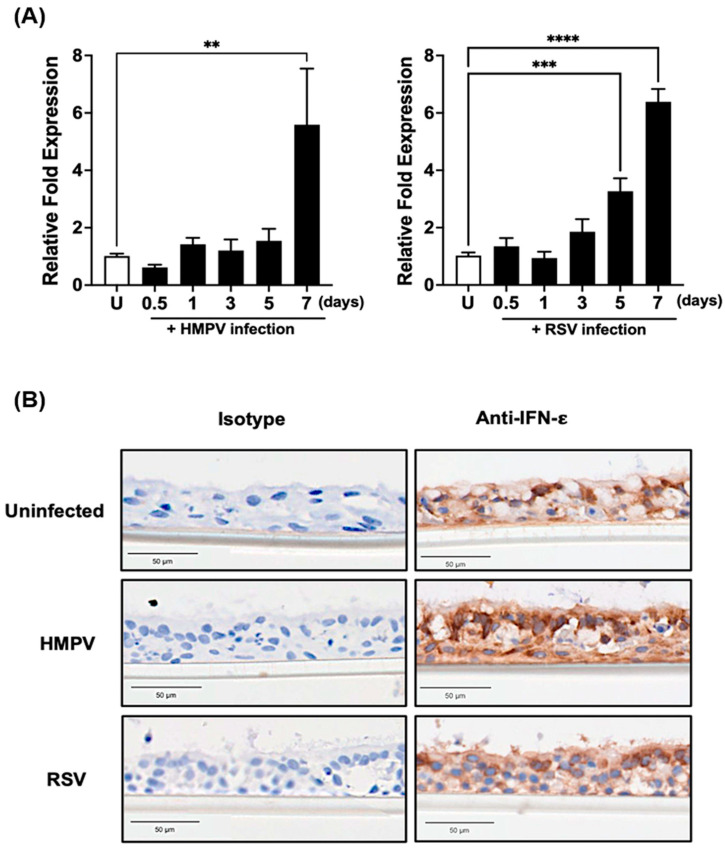
Induction of IFN-ε in primary bronchial epithelial cells. NHBE cells were grown and differentiated in ALI conditions. ALI-cultured cells were infected with HMPV or RSV at an MOI of 0.02. Cell lysates were collected at 0.5, 1, 3, 5, and 7 days after infection and analyzed for the expression of IFN-ε by (**A**) RT-qPCR. The bar graphs represent the mean ± standard errors of the means (n = 3 donors). Statistical differences were calculated using ANOVA followed by a Sidak’s multiple comparison ** *p* < 0.01; *** *p* ≤ 0.001; **** *p* ≤ 0.0001; and (**B**) Immunohistochemistry. Cells were fixed at day 7 after infection and analyzed for the expression of IFN-ε. Bar = 50 μm. Uninfected (U).

To investigate the antiviral effect of IFN-ε in a system that better resembles the human airway epithelium, we used a pseudostratified tissue of NHBE cells cultured under ALI conditions. First, we characterized the IFN-ε response induced by RSV and HMPV. Cells were infected with HMPV or RSV at an MOI of 0.02, and the expression of IFN-ε was assessed at 0.5, 1, 3, 5, and 7 days after infection. As shown in Figure 6A, HMPV induced the expression of IFN-ε by day 7 after infection with a 5.5-fold induction compared to uninfected cells. RSV induced the expression of IFN-ε as early as 5 days after infection with a 3.2-fold increase relative to uninfected cells, reaching a 6.3-fold increase by day 7. To further characterize the IFN-ε response in ALI cultures, infected and uninfected cells were stained at day 7 for the presence of IFN-ε. Day 7 after infection was chosen based on the data observed in 6A, where the highest expression of IFN-ε was observed at that time. Figure 6B shows that IFN-ε is constitutively expressed in uninfected cells. That expression was increased after HMPV and RSV infection, where we observed that the strongest signal is expressed in the apical side of the culture. No signal was detected when the isotype control antibody was used for cell staining.

### 3.6. HMPV and RSV Are Susceptible to the Antiviral Effect of IFN-ε in Human Bronchial Epithelial Cells

**Figure 7:**
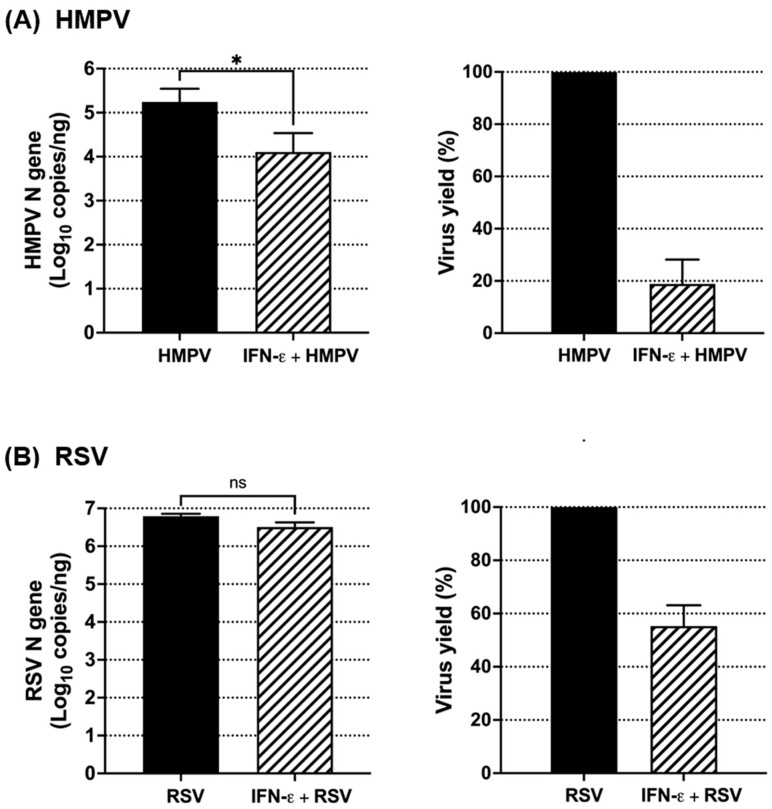
Effect of IFN-ε on RSV and HMPV infection in primary bronchial epithelial cells. NHBE cells were grown and differentiated in an air liquid interface (ALI). Cells were treated with 250 ng/mL of rhIFN-ε for 24 h followed by infection with (**A**) HMPV or (**B**) RSV at an MOI of 0.02 for 3 days. Viral load was assessed by RT-qPCR to quantify the absolute number of viral copies. Virus yield is expressed as a percentage relative to the untreated infected cells. The bar graphs represent the mean ± standard errors of the means (n = 4 donors). Statistical differences were calculated using a student *t*-test. * *p* < 0.05. Non-significant (ns).

To evaluate the antiviral effect of IFN-ε in primary human epithelial cells, NHBE cells from four donors were differentiated into a pseudostratified epithelium through ALI culture. Differentiated cells were treated with rhIFN-ε at a concentration of 250 ng/mL, which was selected based on the antiviral effect observed in A549 alveolar epithelial cells (Figure 4). After 24 h of treatment, the cells were infected with HMPV or RSV at an MOI of 0.02 for 3 days. Cell lysates were collected and analyzed for their viral content by quantifying the number of viral copies. As shown in Figure 7A, the treatment with IFN-ε decreased the HMPV load by 1.1 log10, reducing the viral yield to 18.8%. Regarding RSV infection, we observed that although the virus was sensitive to the effect of IFN-ε, the effect was marginal. The data in Figure 7B show that the RSV load was reduced by 0.2 log10, resulting in a decreased virus yield of 55.2% compared to untreated cells. Together, these data suggest that HMPV is more susceptible to the antiviral effects of IFN-ε, in primary airway human epithelial cells, than RSV.

## 4. Discussion

Although RSV and HMPV are pneumoviruses that share multiple characteristics, their IFN response is differentially induced in vitro and in vivo [40,49,50,51,52]. Type I IFNs, particularly α and β, are known to control the viral replication of RSV and HMPV in both in vitro and in vivo models [39,53,54,55,56]. Nonetheless, the roles of other members of the type I IFN family, such as IFN-ε, are less characterized. IFN-ε shares a 30% amino acid homology with IFN-α and IFN-β [57] and has the ability to signal through IFNAR1 and IFNAR2 [6,58]. IFN-ε has been reported to control the infection of HIV [10,11], ZIKV [4,9,59], and HSV [3,6,60]. However, to the best of our knowledge, this is the first report to determine the antiviral role of IFN-ε in respiratory viral infections.

It is well known that airway epithelial cells are the main target of the viral replication for HMPV and RSV [51,61]. Therefore, we used a model of epithelial cells to study the antiviral role of IFN-ε in these pneumovirus infections. When analyzing the IFN response in A549 cells, we found that both RSV and HMPV induced similar levels of IFN-ε expression during the first 48 h. However, at 72 h, we observed a decrease of IFN-ε in HMPV-infected cells, while in cells infected with RSV, the IFN-ε expression was sustained. A similar effect was observed for IFN-α. The mechanisms responsible for the observed differences in IFN-ε and IFN-α between the two viruses at 72 h are unknown and likely multifactorial. However, it has been reported that the small hydrophobic (SH) protein of HMPV inhibits STAT1 phosphorylation [62,63], which could contribute to a reduced ISGF3 activation and decreased positive feedback loop necessary to induce the expression of IRF7 and IFN-α at a later phase [64]. A similar mechanism could apply to IFN-ε induced in HMPV-infected cells. Overall, future research is needed to understand the molecular mechanisms involved in the induction of IFN-ε by respiratory viral infections in epithelial cells. Finally, when the expression of IFN-ε was assessed in ALI organotypic airway tissue cultures using NHBE cells, the expression of IFN-ε was induced by both viruses at about a sixfold increase compared to uninfected cells. Similar transcript levels have been observed in nasopharyngeal samples from Severe Acute Respiratory Syndrome Coronavirus-2 (SARS-CoV-2)-infected individuals [65].

The induction of the IFN response by viral infections is initiated by the activation of PRRs [66]. The activation of RIG-I [67,68,69], MDA5, and TLRs [70,71,72,73,74,75,76,77,78] has been documented for RSV and HMPV. RIG-I and MDA5 are cytosolic receptors that can bind 5′pppRNAs, short dsRNA, and long dsRNA [45]. TLRs can be found as transmembrane receptors on the cell surface or the endosomal compartment [79] to recognize multiple molecules including ssRNA, dsRNA, or viral proteins [80]. TLRs can further activate the adaptor molecule MyD88 to propagate downstream TLRs signaling pathways [81]. Here, we demonstrate that HMPV and RSV induce the expression of IFN-ε, mostly through RIG-I, suggesting that 5′pppRNAs or dsRNAs (<300 bp) should be generated during the viral infection to induce the expression of IFN-ε. The contribution of MDA5 during RSV infection was also noted, which suggests that long dsRNAs (>2 kb) may also play a role in the induction of IFN-ε by RSV. The role of TLRs in the induction of IFN-ε appears to be marginal since no significant change was observed in the expression of IFN-ε in MyD88 KO-infected cells.

Once secreted, IFNs induce the antiviral state by activating signaling pathways that induce the expression of ISGs to dampen viral replication [48]. Multiple ISGs are induced after the IFNs bind to their receptors and mainly trigger the JAK/STAT signaling pathway [82]. ISGs are the effector molecules that control viral replication through diverse molecular mechanisms [82,83]. To characterize the ability of IFN-ε to induce ISGs in airway epithelial cells, A549 cells were treated with increasing concentrations of rhIFN-ε. Our findings in Figure 3 indicate that IFN-ε induces an antiviral gene signature in respiratory cells, including IFIT1, IFIT2, IFIT3, ISG15, and MX1, with ISG15 predominantly induced at a lower concentration than the other ISGs analyzed, suggesting that ISG15 is a major effector of the antiviral activity of IFN-ε. ISG15 can interfere with viral replication through several mechanisms, including the ISGylation of viral proteins and the inhibition of viral egress [84]. In line with our findings, the induction of ISG15 by recombinant IFN-ε has also been observed in the ectocervical cell line Ect1 [4], which is relevant to ZIKV infection since ISG15 is known to be one of the key ISGs that protect against ZIKV [85]. The antiviral effect of ISG15 against RSV has been reported in in vitro experiments using A549 cells [86]. However, further research on the antiviral effect of ISG15 and other ISGs on HMPV replication is warranted.

In order to investigate the antiviral effect of IFN-ε on RSV and HMPV infection, we first determined the viral susceptibility of these viruses using A549 cells. Several studies have demonstrated the efficacy of IFN-ε as a potential therapeutic agent in viral infections of the human genital tract, but no investigation has been conducted so far regarding their antiviral effect in the respiratory tract. Our findings in Figure 4 indicate that the treatment of A549 cells similarly reduced the percentage of infected cells for both viruses, suggesting a comparative antiviral effect of IFN-ε against RSV and HMPV. To our knowledge, we present here the first evidence that HMPV and RSV infections are sensitive to the effect of IFN-ε. Furthermore, we observed that IFN-ε also interferes with the productive infection of RSV and HMPV since the viral titers in the supernatants of the infected cells were also reduced. However, we observed that the viral titer of HMPV was more affected than that of RSV, suggesting a differential effect of IFN-ε on viral replication for these respiratory viruses.

The data obtained in A549 cells were further validated using primary human bronchial epithelial (NHBE) cells differentiated to a pseudostratified epithelium, representing a more physiological airway model for respiratory infections [87]. Although IFN-ε, in NHBE cells, reduced the number of viral copies of both viruses, HMPV was more susceptible to IFN-ε than RSV, resembling the effect we observed in Figure 5 using A549 cells. The differential response of RSV and HMPV to IFN-ε is currently unknown and warrants further research. However, it is known that several RSV proteins, such as the nonstructural proteins 1 and 2 (NS1, NS2) and the surface attachment protein (G), alter the IFN responses [88]. Moreover, it has been reported that NS2 protein from RSV can impair protein ISGylation. For instance, the binding of NS2 to Beclin 1 hindered Beclin 1 ISGylation, promoting pro-viral autophagy in RSV-infected cells [89]. Nevertheless, whether the NS proteins of RSV interfere with the IFN-ε-induced ISGs to promote viral replication has yet to be investigated.

Furthermore, our current findings show that IFN-ε is constitutively expressed in NHBE cells in an organotypic culture resembling the characteristics of the lining of the bronchioles. This observation correlates with data from non-human primates and mice, where the expression of IFN-ε in the lining of the bronchioles has been reported [7,8]. Furthermore, the basal expression of IFN-ε in human nasal washes indicates that IFN-ε expression is higher in children than in adults [65]. The variable susceptibility of viral infections to IFN-ε has been observed in other viruses such as HIV, ZIKV, and HSV infection [3,4,6,9,10,11,59,60]. In the context of respiratory viral infections, the data are very limited. However, it has been reported that IFN-ε is induced in SARS-CoV-2 positive patients [65]. Overall, these findings highlight the need to further investigate the role of IFN-ε in combating respiratory viruses, particularly when it appears that not all respiratory viruses are equally susceptible to the antiviral effect of IFN-ε, as we observed in this work.

The ability to modulate the immune response and inhibit viral replication makes IFNs important molecules for treating respiratory viral infections. The current clinical trials with type I and III interferons to treat respiratory viral infections [90] make IFNs a potential option to treat or prevent lower respiratory tract infections caused by viral pathogens. That is relevant since there is a lack of RSV and HMPV preventive vaccines for children. Therefore, the use of IFN-ε in the respiratory tract could leverage its ability to induce a robust antiviral state, offering a targeted and efficient approach to treating respiratory viral infections.

## 5. Conclusions

IFN-ε is a critical component of the innate immune response in different mucosal tissues. We have reported for the first time that IFN-ε can reduce the infection and replication of RSV and HMPV in human epithelial cells, observing that HMPV was more susceptible to the antiviral effects of IFN-ε than RSV. These results highlight the relevance of IFN-ε in the respiratory tract and the need to explore its antiviral effect against other respiratory viral infections. While the antiviral therapeutic potential of IFN-ε is clear, more research is needed to fully elucidate its role in the respiratory tract and harness its protective properties for future clinical applications.

## Data Availability

The data supporting the conclusions of this research manuscript are all present within the article.

## Supplementary Materials

The following supporting information can be downloaded at: https://www.mdpi.com/article/10.3390/vaccines12101198/s1, Western blots analysis of Figure 2A.
