# Supplementary material for: Interferon Epsilon-Mediated Antiviral Activity Against Human Metapneumovirus and Respiratory Syncytial Virus
Source: Vaccines (Basel). 2024 Oct 21;12(10):1198. doi: 10.3390/vaccines12101198 (PMC11511582; doi:10.3390/vaccines12101198)
Supplement: Supplementary file 1 [file vaccines-12-01198-s001.zip › vaccines-3239280-supplementary.pdf]

Original Image for Figure 2

Western blot gels images for RIG-I KO cells.

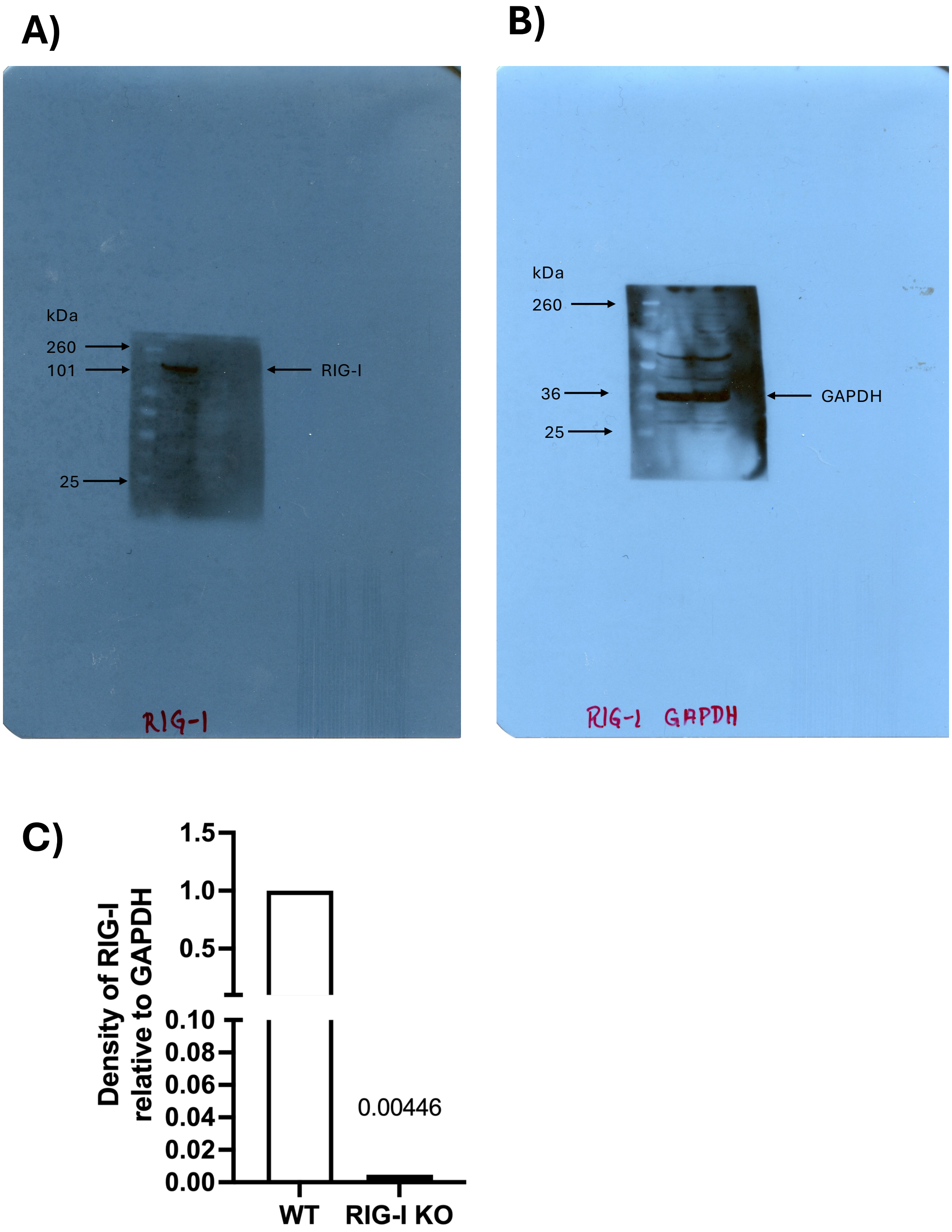

SDS electrophoresis and Western blotting of RIG-I KO A549 cells. **(A)** Gel blotted using an anti-RIG-I antibody. **(B)** The same gel was stripped and stained with an anti-GAPDH antibody. Molecular weights ran from 260 to 25kDa. **(C)** Densitometry analysis of protein expression to the loading control.

Original Image for Figure 2

Western blot gels images for MDA5 KO cells.

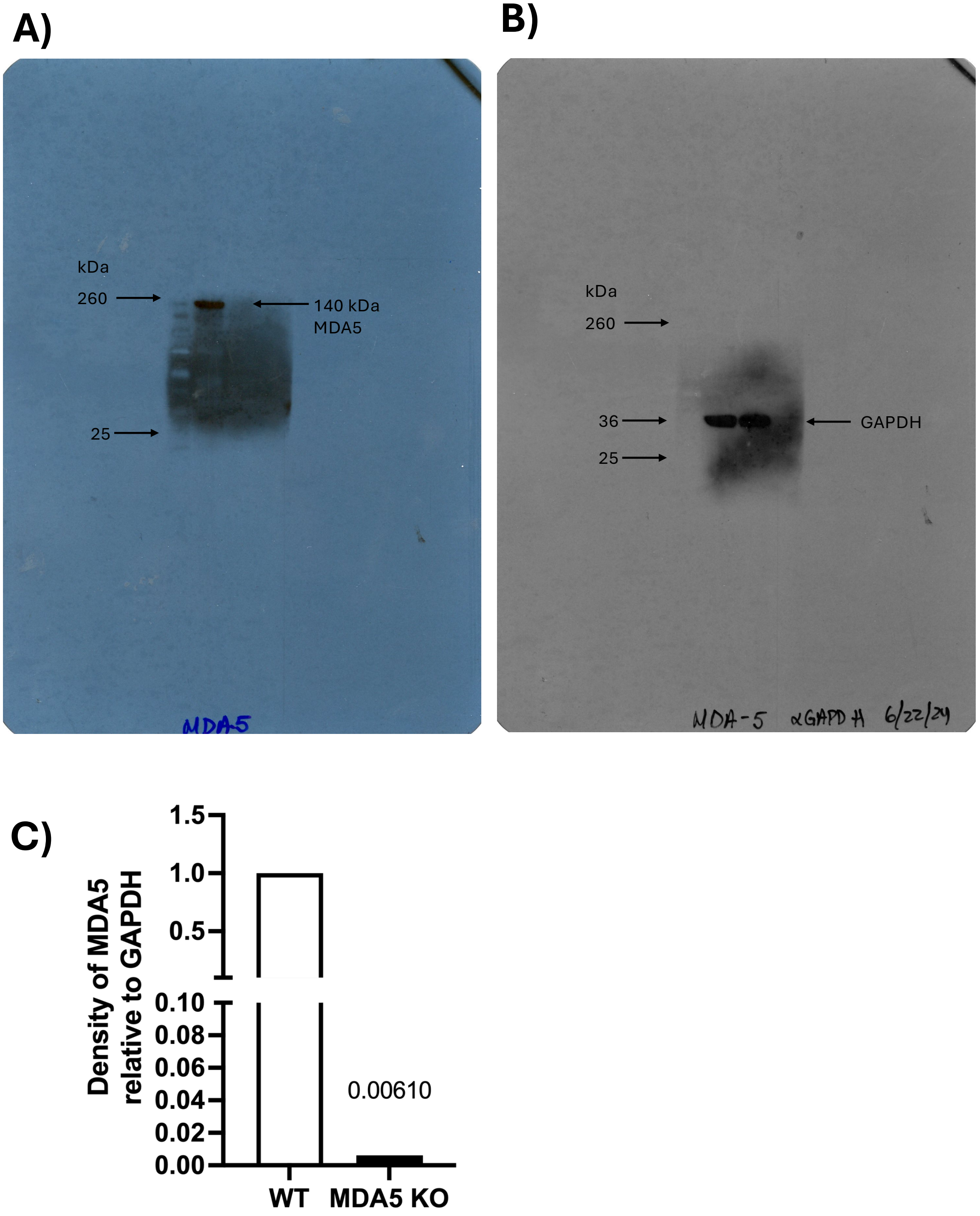

SDS electrophoresis and Western blotting of MDA5 KO A549 cells. **(A)** Gel blotted using an anti-MDA5 antibody. **(B)** The same gel was stripped and stained with an anti-GAPDH antibody. Molecular weights ran from 260 to 25kDa. **(C)** Densitometry analysis of protein expression to the loading control.

Original Image for Figure 2

Western blot gels images for MyD88 KO cells.

A) **B)**

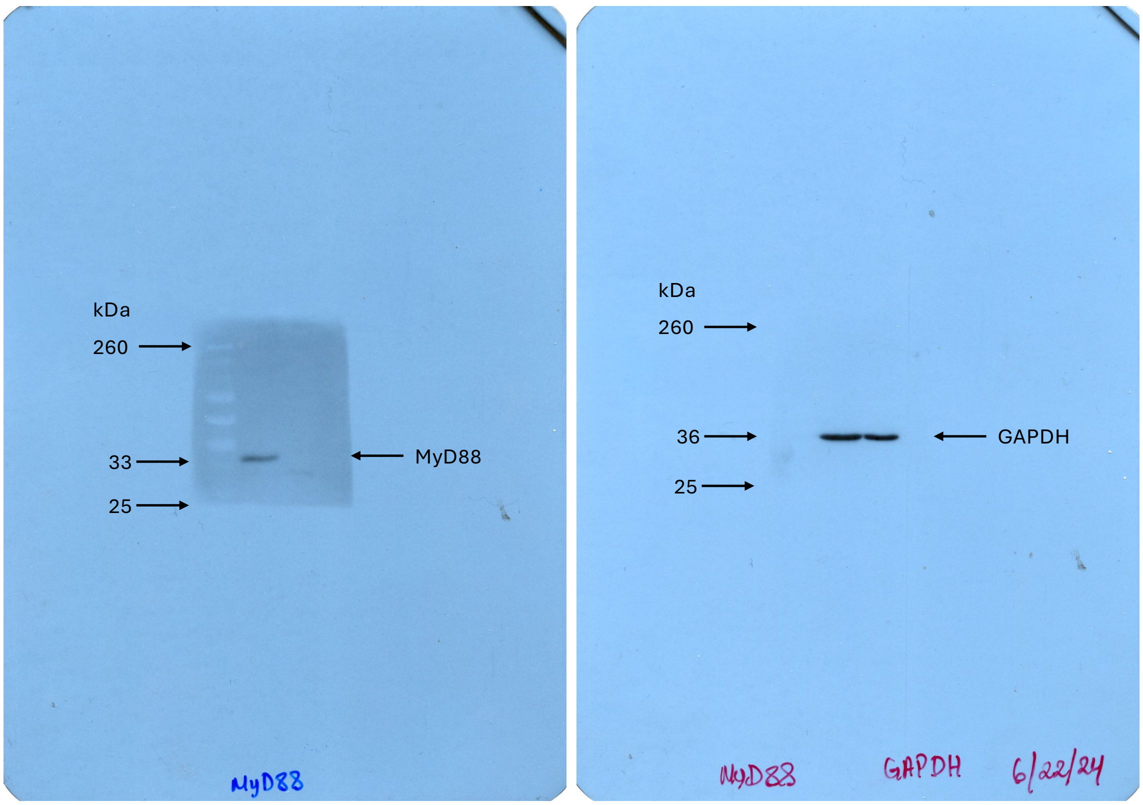

C)

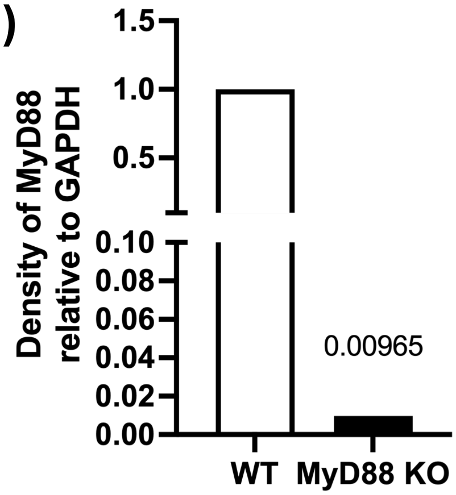

SDS electrophoresis and Western blotting of MyD88 KO A549 cells. **(A)** Gel blotted using an anti-MyD88 antibody. **(B)** The same gel was stripped and stained with an anti-GAPDH antibody. Molecular weights ran from 260 to 25kDa. **(C)** Densitometry analysis of protein expression to the loading control.
